# Supplementary material for: Patterns of Intron Gain and Loss in Fungi
Source: PLoS Biol. 2004 Nov 30;2(12):e422. doi: 10.1371/journal.pbio.0020422 (PMC532390; doi:10.1371/journal.pbio.0020422)
Supplement: Table S1 — Also available at http://genes.mit.edu/NielsenEtAl/. (4.3 MB ZIP). [file pbio.0020422.st001.zip › NielsenEtAl/html/1420.html]

AN3591.1.NCU09266.1.MG01606.1.FG00490.1


```
 CLUSTAL W (1.82) Multiple Sequence Alignments - Introns Inserted


Sequence 1: MG01606.1	543 aa
Sequence 2: FG00490.1	559 aa
Sequence 3: NCU09266.1	545 aa
Sequence 4: AN3591.1	536 aa
Alignment Length: 561 aa
Number Identitical Residues: 374 aa
Alignment Score (without introns) 16395


MG01606.1 	---------------MASSQSGS---AARRLQATAQQLRPIASAAMASTPTNYPTTHEKI
NCU09266.1	---------------MSSPAKTPSATAARRIHATANHLRPAA-AALESTATSYPTTHDKI
FG00490.1 	MGASSRSTPALSRASILTSSSGPSHVAARRIHATTKQLQPVT-AALASTASSYPTTHAKV
AN3591.1  	------------------------MAALRRLHATAQQFAPST-TSAASTATEYPTTHEAI
          	                          * **::**:::: * : ::  **.:.*****  :

MG01606.1 	TEPVDTPYFIDNKFIASEATSFIDLHDPATNNLVTRVPQMTDAELKAAVASAEKAFPAWR
NCU09266.1	ADVKDTPWFIDNKFVTSSTTQYIDLHDPATNNLVTRVPQNTDEELKAAVASAQKAFESWR
FG00490.1 	-EVVDTPYFIDNKFVASTADKYIDLHDPATNELVTRVPQMTDAEMKAAVESAEKAFKSWK
AN3591.1  	ANPIDTTNFLNNEFVPSKASTWIDLYDPATNNLVTRVPQSTDEELRAAVEAAQKAFPAWR
          	::  **. *::*:*:.* :  :***:*****:******* ** *::*** :*:*** :*:

MG01606.1 	ATSVLARQQIMFKFVALIRENWDRLAASITLEQGKTFADAKGDVLRGLQVAEAACAAPEL
NCU09266.1	NTSVLHRQAIMFKFVGLIRENWDRLAASITLEQGKTFADAKGDVLRGLQVAEAACAAPEL
FG00490.1 	NTSVISRQQIMFRFVQLIRENWDRLAASITLEQGKTFADAKGDVLRGLQVAEAAVAAPEL
AN3591.1  	ATSIMARQQIMFKFVNLIRANWDRLAASITLEQGKTFADAKGDVLRGLQVAETACGITTQ
          	 **:: ** ***:** *** ********************************:* . .  

MG01606.1 	LKGEVLEVAKDMETRTYREPLGVVAAICPFN1FPAMIPLWCIPIATITGNTLILKPSERD
NCU09266.1	LKGEVLEVAKDMETRSYRDPLGVVAAICPFN~FPAMIPLWCIPIATVTGNTLILKPSERD
FG00490.1 	LKGEVLEVAKDMETRTYREPLGVTAAICPFN1FPAMIPLWCIPIATITGNTLILKPSERD
AN3591.1  	ITGEVLEVAKDMETRSYREPLGVVAAICPFN1FPAMIPLWCIPIATITGNTMVMKPSERD
          	:.*************:**:****.******* **************:****:::******

MG01606.1 	PGAAMILAELCQKAGFPDGVVNIVHGAHNTVNFILDDPAIKAISFVGGNKAGEYIFSRGS
NCU09266.1	PGAAMILAELVQKAGFPEGVVNIVHGAHRTVNFILDEPAIKAISFVGGNKAGEYIFARGS
FG00490.1 	PGAAMIIAELVEKAGFPAGVVNIIHGAHRTVDFILDEPAIKAISFVGGNKAGEYIFSRGS
AN3591.1  	PGAAMILAELAREAGFPPGVINIIHGSAKTVDFILDAPEIKAISFVGGNRAGEYIYTRGS
          	******:*** .:**** **:**:**: .**:**** * **********:*****::***

MG01606.1 	ANGKRVQANLGAKNHAAVLPDCNKNQFLNAIVGAAFGAAGQRCMALSTLVMVGETKEWLP
NCU09266.1	ANGKRVQANLGAKNHAAVLPDCNKNQFLNAVVGAAFGAAGQRCMALSTLVMVGETKNWLP
FG00490.1 	ANGKRVQANLGAKNHAVVSPDANKNQFINSIVGAAFGAAGQRCMALSTLVMVGETKEWLH
AN3591.1  	ANGKRVQANLGAKNHAAVLPDANKNQTINAIVGAAFGAAGQRCMALSTLVTVGETKEWLP
          	****************.* **.**** :*::******************* *****:** 

MG01606.1 	ELAELAKGLSVNGGFEEAADLGPVISPQSKERIESLIASAEKEGAKILLDGRGFAPSKYP
NCU09266.1	ELAERAKALNVNGGFEEGADLGPVISPQSKERIESLIASAEQEGATILLDGRGYKPAKYP
FG00490.1 	DVAEQAKNLNVNGGFEQGADLGPVITPQSKERIEKLIDSAEKEGATILLDGRGFKPSKYP
AN3591.1  	EMAERAKALNVNGGFEEGADLGPVISPESKKRIEDLIASAEEEGATILLDGRGYKPEKYP
          	::** ** *.******:.*******:*:**:***.** ***:***.*******: * ***

MG01606.1 	NGNWVGPTIITDVTKDMECYKQEIFGPVLVCLNVETLDDAVELINSNEYGNGVAIFTRSG
NCU09266.1	NGNWIAPTIISNVTPSMKCYREEIFGPVLVCLNVETLDEAIELINSNEYGNGVAIFTRSG
FG00490.1 	NGNWVGPTIITNVTPDMTCYKEEVFGPVLVCLNAESIEDAIDLVNKNEYGNGTAIFTRSG
AN3591.1  	NGNFIGPTIITGVTPGMKCYKQEIFGPVLVCLEVETLDDAIELINKNEYGNGAAIFTRSG
          	***::.****:.** .* **::*:********:.*::::*::*:*.******.*******

MG01606.1 	ATAEKFRRNIEAGQVGINVPIPVPLPMFSFTGNKKSVAGGGASTFYGKPGINFYTQLKTV
NCU09266.1	PTAETFRRRIEAGQVGINVPIPVPLPMFSFTGNKKSIAGGGASTFYGKPGVNFYTQLKTV
FG00490.1 	ATAEIFRKNIEAGQVGINVPIPVPLPMFSFTGNKKSIAGGGANTFYGKPGINFYTQLKTV
AN3591.1  	PTASRFQKDIEAGQVGINVPIPVPLPMFSFTGNKKSIAGGGANTFYGKPGLQFYTQQKTV
          	.**. *:: ***************************:*****.*******::**** ***

MG01606.1 	TALWQSADAISKKADVAMPTHS
NCU09266.1	TAHWAAQDAIGQKADVAMPTHS
FG00490.1 	TALWQSADAVAKKADVSMPTQQ
AN3591.1  	TSLWRAEDAVSTKAHVVMPTHS
          	*: * : **:. **.* ***:.
```
